# The interplay of Criterion A of the Alternative Model for Personality Disorders, mentalization and resilience during the COVID-19 pandemic

**DOI:** 10.3389/fpsyg.2022.928540

**Published:** 2022-07-25

**Authors:** Jeff Maerz, Anna Buchheim, Luna Rabl, David Riedl, Roberto Viviani, Karin Labek

**Affiliations:** ^1^Institute of Psychology, University of Innsbruck, Innsbruck, Austria; ^2^University Hospital of Psychiatry II, Medical University Innsbruck, Innsbruck, Austria; ^3^Department of Psychiatry and Psychotherapy III, University of Ulm, Ulm, Germany

**Keywords:** mentalization, Criterion A, personality functioning, resilience, depression, life satisfaction, COVID-19, mental health

## Abstract

**Background and aims:**

The COVID-19 pandemic has been accompanied by a worsening of mental health levels in some, while others manage to adapt or recover relatively quickly. Transdiagnostic factors such as personality functioning are thought to be involved in determining mental health outcomes. The present study focused on two constructs of personality functioning, Criterion A of the Alternative Model for Personality Disorders (AMPD, DSM-5) and mentalization, as predictors of depressive symptoms and life satisfaction during the COVID-19 pandemic. A second focus of the study was to examine whether this relationship was mediated by resilience.

**Methods:**

Linear regression analyses were used to examine the relationship between personality functioning measured by Criterion A (AMPD, DSM-5) and mentalizing abilities as predictors, and depression and life satisfaction as mental health outcomes. To assess the hypothesis that this relationship is mediated by resilience a structural equation modeling approach was conducted. Data from *N* = 316 individuals from the general population were collected.

**Results:**

Linear regression models revealed highly significant associations between Criterion A/mentalization and both outcome measures. Structural equation models showed a significant partial mediation by resilience of these relationships.

**Conclusion:**

Our results support the hypothesis that mentalizing serves as a protective function by promoting resilience to the impact of stress and threats. Criterion A and mentalization performed similarly as predictors of mental health outcomes, providing empirically overlapping operationalizations of personality functioning. This finding emphasizes the importance of personality functioning in positive and negative mental health outcomes. Furthermore, our results are consistent with a mediating role of resilience.

## Introduction

Humans have had to adapt to disasters, trauma, adversity, threats, and other significant life stressors. Although the COVID-19 pandemic has directly or indirectly affected almost everyone worldwide, recently published data showed, that most individuals from non-clinical populations remained mentally healthy (Ahrens et al., 2021). Beside the profound impact of the COVID-19 pandemic on people’s lives, there has been growing interest in evaluating the existence of potential protective and risk factors to mental health during the pandemic (Ahrens et al., 2021; Engert et al., 2021; Veer et al., 2021), since the same factors may be generally relevant as predictors of mental health (Rutten et al., 2013; Masten, 2019).

Public health research has been drawing attention to the importance of the severity of personality disorders (PD) for mental health, where “severity” refers here not only to a clinical condition but is meant to describe functioning at various levels in the healthy or non-clinical population as well (Tyrer and Johnson, 1996; Yang et al., 2010; Bender et al., 2011; Waugh et al., 2017; Bender, 2019; Tyrer et al., 2019). Personality functioning is generally assessed within a dimensional model as the Criterion A of the alternative model of personality disorders (AMPD in DSM-5; Bender et al., 2011; Skodol, 2012; American Psychiatric Association, 2013) and as level of severity in the ICD-11 (Tyrer et al., 2011, 2015). Studies on personality in recent years have shown that deficits in personality functioning are the most important vulnerability factor for psychosocial dysfunction and the development of mental disorders (Hopwood et al., 2011; Morey, 2017; Tyrer et al., 2019; Buer Christensen et al., 2020), suggesting a negative role in personal resilience.

Another construct related to personality functioning is mentalization, the capacity to elaborate on other people’s state of mind and intentions (Bateman and Fonagy, 2004; Luyten et al., 2020). Mentalization was found to overlap conceptually and operationally with Criterion A (Bender et al., 2011; Zettl et al., 2020; Rishede et al., 2021). Furthermore, fostering social cognition competences by improving mentalization capacities is a common element in all therapies of borderline personality disorder (BPD; Bateman and Fonagy, 2004; Viviani et al., 2011; Fischer-Kern et al., 2015; Buchheim and Diamond, 2018; Fonagy et al., 2019; Labek et al., 2019; Lüdemann et al., 2021). Moreover, it has been shown that mentalization capacity may provide a possible alternative assessment of personality functioning (Zettl et al., 2020). Accordingly, previous research has revealed that high levels of mentalization capacity may be used as predictors or protective factors of better mental health outcomes in times of stress (Hayden et al., 2019; Schwarzer et al., 2021a,b). There are only few studies that examined associations between mentalizing and distress or pathologies in healthy individuals and patients during the pandemic (Lassri and Desatnik, 2020; Poulios et al., 2021; Kvarstein et al., 2022; Yatziv et al., 2022). Furthermore, some pre-pandemic studies have demonstrated a mediating protective influence of mentalizing capacity (Hayden et al., 2019; Poulios et al., 2021; Schwarzer et al., 2021a,b). Previous studies have separately investigated the association of Criterion A and mentalizing with depressiveness and life satisfaction (e.g., Ballespí et al., 2018, 2021; Borelli et al., 2019; Luyten et al., 2019).

Diagnostic systems for PD such as the AMPD or ICD-11 are well suited to current conceptualizations of psychological resilience, which adopt a transdiagnostic framework (Kalisch et al., 2015; Feldman, 2021) and embrace the paradigm-shift toward dimensional diagnoses of clinical diagnostic systems when investigating factors and mechanisms, that enable human psychological functioning despite substantial hardship (Fonagy et al., 2017a). Based on the Appraisal Theory of Resilience (Kalisch et al., 2015). Fonagy et al. (2017a) convincingly integrated resilience-promoting qualities of mentalization in a broader context. By considering BPD, the authors expanded their understanding and conceptualization of PD into a resilience framework in which PDs are not characterized as impairments, but as the absence of resilience and social-communicative flexibility (Fonagy et al., 2017a,b). In this view, social communication with significant attachment figures, but also within important individuals in the social system, constitute the basis for learning how to mentalize and for promoting resilience (Bateman et al., 2018; Fonagy and Campbell, 2021). However, the capacity to mentalize can be impaired or even collapse under a variety of circumstances. While mentalizing capacities can be quite accurate under conditions of low arousal, they can be impaired under stressful situations and conditions of high arousal, e.g., when facing threat or loss (Luyten and Fonagy, 2015). This is of particular importance, because recent studies have provided preliminary evidence that the pandemic can restrict mentalization capacity in both healthy individuals and patients due to high levels of stress and the dramatic change in social interactions during lockdown periods (Lassri and Desatnik, 2020; Ventura Wurman et al., 2021; Yatziv et al., 2022).

In the current study we aimed to improve our understanding of the interplay between personality functioning (Criterion A and mentalization) on the one hand and depressive symptoms and life-satisfaction on the other hand in the context of the COVID-19 pandemic. Along with other pre-pandemic studies (Crempien et al., 2017; Fischer-Kern and Tmej, 2019; Veenstra et al., 2022) we expected an association between Criterion A/mentalization scores with depressive symptoms and life satisfaction in our sample during the COVID-19 pandemic. Beyond these expected findings, we were specifically interested in verifying whether Criterion A and mentalization behave similarly as predictors of mental health outcomes. A second aim of the study was to test the hypothesis by Fonagy et al. (2017a), that the protective effect of mentalization capacity against affective symptoms such as depressiveness is mediated by resilience. We also hypothesized that in case mentalization and Criterion A largely overlap, as argued by Zettl et al. (2020), the same mediation model would be observed with mentalization capacity as a predictor instead of Criterion A. Finally, these regression and mediation models should provide similar patterns of association, but reversed in sign when tested on a positive mental health outcome, such as life satisfaction.

The headings of this paper are organized as follows: In the remaining introductory part, we will explain the constructs of Criterion A and mentalization adopted in the present work in the light of the current literature. After providing details on the sample and the instruments in the Methods section, we will report the results on the expected associations between Criterion A/mentalization and our two outcome measures. Structural equation models will further investigate the mediation role of resilience on these two outcomes, irrespective of whether personality functioning was measured by Criterion A or mentalization capacity. In the discussion, we will summarize the implications of these findings for the dimensional characterization of personality functioning as a mental health predictor and point to some important limitations of the present study.

### Criterion A and mentalization

Criterion A (personality functioning) in DSM-5, at its core, is characterized by basic psychological human capacities in the domains of self (identity, self-direction) and interpersonal relationships (intimacy, empathy; DSM-5, American Psychiatric Association, 2013; ICD-11, World Health Organization [WHO], 2019; see Caligor and Kernberg, 2005; Bender et al., 2011; Tyrer et al., 2019). Criterion A is generally aligned with broad psychodynamic concepts (Bender et al., 2011; Blüml and Doering, 2021), characterized as personality organization or structure of the representations of self and others (Kernberg, 1967; Bender et al., 2011; Skodol et al., 2011; Pincus et al., 2020; Blüml and Doering, 2021). Both mentalization and Criterion A attempt to assess the quality of representations as internal mental states of self and others that are considered fundamental to building healthy social relationships and enable adaptations to the social environment (Fonagy and Luyten, 2018; Fonagy et al., 2018; Luyten et al., 2020). Impairments in personality functioning have been associated with emotional dysregulation, poor impulse control, and lower achievement of long-term goals (Fonagy and Target, 1997; Dimaggio et al., 2006; Bender et al., 2011; Bateman and Fonagy, 2015; Euler et al., 2019). In contrast, individuals with high scores in personality functioning were able to understand their own interactions with others, enter long-term and fruitful collaborations, care for others, demonstrate empathy, and establish and maintain stable interpersonal relationships (Parker et al., 2002; Bender et al., 2011).

Although both Criterion A and mentalization have their origins in the field of psychopathology, they encompass “optimal functioning” at the healthy extreme of the assessment score. This is key for understanding the positive effect in mental health in the general population and quality of life (Esposito et al., 2020) or well-being (Stein, 2006; Fonagy et al., 2017a; Ballespí et al., 2018, 2021; Borelli et al., 2019; Luyten et al., 2020; Schwarzer et al., 2021a,b). At the other extreme, personality functioning provides an assessment of severity of impairment, reflecting the increasing theoretical and empirical consensus that PD can be understood along a severity continuum (Hopwood et al., 2011; Morey et al., 2011). Accordingly, low personality functioning is found in multiple disorders, including anxiety disorders and depression (e.g., Fonagy and Bateman, 2007; Busmann et al., 2019; Fischer-Kern and Tmej, 2019; Luyten et al., 2019, 2020; Buer Christensen et al., 2020; Dagnino et al., 2020; Veenstra et al., 2022).

In this context, research using factor analytic approaches is noteworthy. There is now some evidence for the existence of a general factor of personality disorder (g-PD; Sharp et al., 2015), within a psychopathological model referred to as a “p-factor” (Caspi et al., 2014; Bender, 2019), which is thought to represent self-other pathology as an underlying vulnerability that predisposes any type of psychopathology (Sharp et al., 2015; Fonagy and Campbell, 2021; see recent discussions on the integration of Criterion A on Hierarchical Taxonomy of Psychopathology Bender, 2019; Widiger et al., 2019). At present, no standardized assessment of the p-factor is available, but it appears likely that personality functioning may provide related information. In sum, current findings underline the idea of a common factor supporting a dimensional conceptualization of personality disorder severity (Sharp et al., 2015; Zimmermann et al., 2020), meaning that high scores indicate persistent psychological vulnerability over time and a lack of resilience to life stressors (Fonagy et al., 2017a; Bateman et al., 2018).

## Materials and methods

### Participants

The study took place between February 23 and April 2, 2021, at the Institute of Psychology at the University of Innsbruck (Austria) close in time to the third Austrian lockdown (December 16, 2020 – February 8, 2021). During this lockdown all stores, restaurants, bars, clubs, fitness studios and parks were closed with only few exceptions. Home office was recommended, but not mandatory. Schools and universities applied distance learning, indoor sport and entertainment events were prohibited. Some of these restrictions were successively relaxed on January 18 for individuals who tested negative. At that time, a vaccine against COVID-19 was not yet available for the general population.

The current study is a quantitative cross-sectional online study. The participant sample consisted of students from the University of Innsbruck and their family and friends who gave informed consent to the study. Initially, all students on the campus were invited *via* email to complete the survey and to forward it to their relatives and friends. Overall, 453 participants enrolled in the online survey. After excluding all participants with missing, incomplete, or incorrect data, the dataset included 316 participants. Data were collected in pseudo-anonymized form. Ethical approval was granted by the Review Board for of the Institute of Psychology (N° 34/2020).

### Measures

#### Socio-demographic variables

We assessed the following socio-demographic variables: age, sex, student status, and the social status in childhood. Student status was assessed with a simple Yes/No-question, whereas social status in childhood was assessed with a 10-point Likert scale, ranging from 1 (“bottom social class”) to 10 (“upper social class”).

#### Social connectedness

The UCLA loneliness scale (UCLA LS; Russell et al., 1980) was used in the German version (Döring and Bortz, 1993) to assess social connectedness. The UCLA LS is composed of a 20-item self-report questionnaire in which 10-items are positively and 10-items are negatively worded. The 20 items (e.g., “I feel alone.”) are scored on a 5-point Likert scale from 1 (“I disagree completely”) to 5 (“I agree completely”). The internal consistency (α) of the scale was 0.87. For the present study we chose a 2-factor model (Russell, 1996), with one factor involving alle the negative items and one factor involving all the positive items. For further modeling, we used only the subscale “Social Connectedness,” which includes all the positive items. A higher score represents better social connectedness. We included only social connectedness because of its relevance on resilience in the ongoing pandemic situation (Li et al., 2021).

#### Criterion A

Criterion A was assessed with the Level of Personality Functioning Scale (LPFS-BF; Hutsebaut et al., 2016) in the German version (Spitzer et al., 2021). The LPFS-BF is a brief 12-item self-report questionnaire for assessing Criterion A of the AMPD-system, showing a good internal consistency (α = 0.85). The 12 items (e.g., “I often do not know who I really am.”) are scored on a 4-point Likert scale from 1 (“completely untrue”) to 4 (“completely true”). The LPFS-BF captures two different dimensions, self-functioning, and interpersonal functioning. In this study we inverted all the items for consistency with the terminology “functioning,” resulting in higher scores representing higher personality functioning.

#### Mentalization

For assessing mentalization capacity we used the mentalization questionnaire (MZQ; Hausberg et al., 2012). The scale is composed of 15 items (e.g., “Most of the time it is better not to feel anything.”) on a 5-point Likert scale with a range from 1 (“Do not agree at all”) to 5 (“I fully agree”) and a good internal consistency (α = 0.86). Again, we inverted all the items such that high scores represented higher mentalization capacities.

#### Life satisfaction

We used the Satisfaction with Life Scale (Diener et al., 1985) in its German version as the most commonly applied measure of global life satisfaction (Janke and Glöckner-Rist, 2014). Participants indicated the degree to which they agreed with each of the five items (e.g., “I am satisfied with my life.”) on a 7-point Likert scale, ranging from 1 (“strongly agree”) to 7 (“strongly disagree”). The internal consistency of the scale was good (α = 0.83).

#### Depression symptoms

To examine depression symptoms we included the “Allgemeine Depressionsskala” (ADS; Hautzinger and Bailer, 1993) as a German version of the Center for Epidemiologic Studies Depression Scale (Radloff, 1977) in our battery. This instrument screens for depression symptoms with 20 items (e.g., “I was bothered by things that usually don’t bother me”) on a 4-point Likert scale ranging from 0 (“rarely or at all not”) to 3 (“mostly, the whole time”), with excellent internal consistency (α = 0.92).

#### Resilience

Resilience was measured using the Resilience Scale (RS-13; Leppert et al., 2008) as a German short form of the well-known RS-25 (Wagnild and Young, 1993). With 13 items (e.g., “I usually manage one way or another”) on a 7-points Likert scale, ranging from 1 (“no, do not agree”) to 7 (“yes, totally agree”) this instrument assesses resilience as a combination of the two factors “Acceptance of Self and Life” and “Personal Competence.” The internal consistency of the scale was good (α = 0.89).

### Statistical analysis

We computed Pearson’s correlations to determine whether we find significant association between age, gender, student status, social status in childhood, social connectedness, Criterion A, mentalization, depression symptoms, life satisfaction and resilience. Following Cohen’s (1988) suggestion, we rated Pearson correlation coefficients between variables as small effects (*r* < 0.3), medium effects (0.3 ≤ *r* ≤ 0.5), and large effects (*r* > 0.5).

In preliminary analyses, we examined the data for accuracy of the entries and missing or incorrect values. Participants with missing or incorrect data were excluded.

To test the association between Criterion A and mentalization with depressive symptoms and life satisfaction in our sample, we estimated several separate linear regressions. Age, gender, student status, social status in childhood, and social connectedness were selected as nuisance variables. Criterion A and mentalization were used as predictor variables in separate models. For regression analyses and structural equation modeling (SEM), we report standardized beta values (ß). The linear relations between predictors and residuals, and homoscedasticity were analyzed using scatter plots. Cook’s distance was used to screen for potential influential data points. Multicollinearity was assessed using tolerance (*x* < 0.01) and the variance inflation factor (VIF; *x* < 10).

To test the protective effect of Criterion A and mentalization capacity against affective symptoms such as depressiveness and life satisfaction as mediated by resilience, we used a two-step structural equation modeling approach as recommended by Anderson and Gerbing (1988). In the first step, we applied a measurement model to test whether each of the latent variables were represented by the observed variables. With an acceptable fit to the data of this model, in a second step we estimated a structural model using maximum likelihood. As we didn’t reach normal distribution for the used scales, we divided the items for each scale into three parcels (Hau and Marsh, 2004). The parceling was achieved by assigning each item randomly and without replacement to one of the three corresponding parcels per latent variable (Little et al., 2002).

To evaluate the model fitting, we used the four goodness-of-fit indices suggested by Hu and Bentler (1999): chi-square statistics (*X*^2^), Root-Mean-Square Error of Approximation (RMSEA), Standardized Root-Mean-Square Residual (SRMR), and the Comparative FIT Index (CFI). In accordance with the literature, we define the criteria as followed: RMSEA < 0.06 for a good and <0.08 for an acceptable fitting, SRMR < 0.05 for a good fitting and CFI best if above 0.95 (MacCallum et al., 1996; Hu and Bentler, 1999; Marsh et al., 2004; Schumacker and Lomax, 2016). As suggested in the literature, regarding the chi-square-test we consider a rejection of the null hypothesis (significant *X*^2^) not as a strict non-fitting indicator (Kline, 2005; Steiger, 2007).

Linear regression models were estimated with IBM SPSS (Version 26.0; IBM Corp., 2019). SEM were conducted with IBM SPSS AMOS (Version 26.0; Arbuckle, 2014).

## Results

### Preliminary analysis and descriptive statistics

The age of our sample (*N* = 316) varied from 18 to 68 with a mean age of 26.15 years (*SD* = 10.40). Females represented 65.19% of the sample, while 34.81% of the participants were male. 64.6% of our sample were active students at the time of data collection, while social status in childhood ranged from 1 to 10 with a mean 6.35 (*SD* = 1.63). Descriptive statistics and correlations between all the variables are presented in Table 1.

**TABLE 1:** Descriptive statistics and correlations between age, sex, student status, social status in childhood, social connectedness, Criterion A, mentalization, depressive symptoms, life satisfaction, and resilience.

		*M*	*SD*	1	2	3	4	5	6	7	8	9
1	Age	26.15	10.40									
2	Gender	1.65	0.48	-0.02								
3	Student status	1.35	0.48	0.48**	0.06							
4	Social status childhood	6.35	1.63	-0.06	-0.05	0.02						
5	Social connectedness	3.52	0.46	0.10	-0.04	0.04	0.17**					
6	Criterion A	0.93	0.51	-0.25**	0.10	-0.05	-0.08	-0.50**				
7	Mentalization	3.59	0.68	0.16**	-0.16**	0.04	0.07	0.46**	-0.70**			
8	Life satisfaction	5.06	1.15	0.13*	0.08	0.01	0.15**	0.58**	-0.64**	0.53**		
9	Depressive symptoms	0.84	0.56	-0.18**	0.17**	-0.02	-0.14*	-0.46**	0.65**	-0.56**	-0.60**	
10	Resilience	5.36	0.92	0.12*	-0.05	0.08	0.14*	0.52**	-0.58**	0.48**	0.61**	-0.62**

*M*, mean; *SD*, standard deviation; *p < 0.05, **p < 0.01, n = 316; gender: 1 = male, 2 = female; and student status: 1 = student, 2 = no student.

To compare the scores of our sample with the norm values, we conducted statistical analyses (*t*-tests) for the following measurements: resilience (RS-13, Leppert et al., 2008), depressive symptoms (ADS, Hautzinger and Bailer, 1993), Criterion A (LPFS-BF, Spitzer et al., 2021), mentalization (MZQ, Riedl et al., submitted) and life satisfaction (Glaesmer et al., 2011).

#### Resilience

Examining the RS-13 scale our sample achieved a mean score of *M* = 69.70, (*SD* = 11.91). In direct comparison with a German norm sample, we were not able to find any statistical differences to our study sample [*t*(15) = −0.45, *p* = 0.654]. Criterion A: The LPFS-BF averaged mean resulted in *M* = 23.21, (*SD* = 6.06). When we compare our data with norm values reported by Spitzer et al. (2021), norm values of sample 1 revealed a significantly higher personality impairment in our study sample [*t*(315) = 16.32, *p* ≤ 0.001], whereas no significant statistical difference were found with sample 2 [*t*(315) = 1.44, *p* = 0.150]. Life satisfaction: The results of satisfaction for life (*M* = 25.31, *SD* = 5.77) showed no difference [*t*(315) = 1.32, *p* = 0.189] compared to a German norm sample with 2,519 participants. Depressive symptoms: The ADS norm sample showed a mean sum score of 14.33 (*SD* = 9.66, *n* = 1,205; Hautzinger and Bailer, 1993), which is significant lower than the score reached in our study sample [*M* = 16.80, *SD* = 11.20; *t*(315) = 3.92, *p* < 0.001]. Further, we found in total 123 participants, representing 38,91% of the sample, that exceeded the suggested screening cut-off for depression of *M* = 23. In the norm sample only 17.4% exceeded this cut-off, indicating that our sample included significantly more individuals with depressive symptoms. Mentalization: Inspecting the MZQ, our sample achieved a mean of 2.41 (*SD* = 0.68), which is not significantly different [*t*(315) = −0.44, *p* = 0.661] from a German norm sample (Riedl et al., submitted).

In summary, our analyses indicated that our study sample showed increased depressive symptoms in comparison with a healthy norm sample, similarly to other studies during the pandemic. No other scales showed significant differences with normative values.

### Linear modeling

Because of the strong empirical and conceptual overlap between mentalization and Criterion A (Zettl et al., 2020), we decided to separate both variables for further modeling, expecting to obtain similar results if these two measures really represent mostly the same construct as asserted in the literature, thus providing a robustness check on our models.

In a first step, to describe the sample’s demographic characteristics, a model was estimated with the variables age, gender, social status in childhood, and student status as independent variables and depressive symptoms and life-satisfaction scores as outcome variables. This analysis indicated that young women (age, ß = −0.23, *p* < 0.001; gender, ß = 0.16, *p* = 0.004), with lower childhood social status (ß = 0.15, *p* = 0.007) showed higher depressiveness scores, while student status (ß = 0.83, n.s.) did not reach any level of significance. However, participants′ answers about life satisfaction revealed a positive association with age (ß = 0.18, *p* = 0.005) and higher childhood social status (ß = 0.17, *p* = 0.003) when they are more satisfied. Women reached only a statistical trend (ß = 0.09, *p* = 0.096) in the association with life satisfaction. No effect was found of student status (ß = −0.08, n.s.).

**FIGURE 1:**
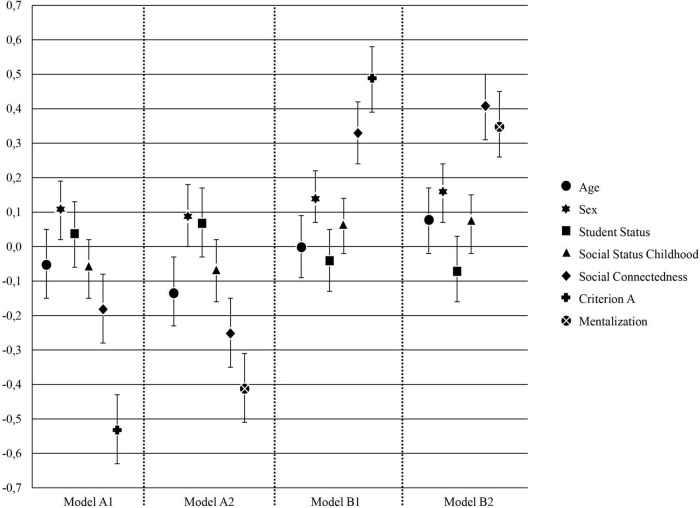
Illustration of standardized regression coefficients β for models A1 and A2 (depressive symptoms) and B1 and B2 (life satisfaction; *n* = 316). Error bars representing 95% bootstrapped confidence intervals.

In a next step, we tested the possible predictive value of Criterion A and mentalization in separate multivariate regression models with depressive symptoms (Model A1 and A2) and life-satisfaction scores (Model B1 and B2) as outcome variables. Each regression model included the covariates age, gender, social status in childhood, student status, and social connectedness. We entered social connectedness as a control variable in the models because it is considered as a relevant factor for resilience and the current pandemic situation (Li et al., 2021). Results are shown in Table 2 and Figure 1.

**TABLE 2:** Summary of linear regression analysis for variables predicting depressive symptoms and life satisfaction.

	Model A1 DS		Model A2 DS		Model B1 LS		Model B2 LS	
	β	95% – CI	β	95% – CI	β	95% – CI	β	95% – CI
Age	-0.05	[-0.15, 0.05]	-0.13	[-0.23, -0.03] *	0.00	[-0.09, 0.09]	0.08	[-0.02, 0.17]
Sex	0.11	[0.02, 0.19] *	0.09	[0.00.18]	0.14	[0.07, 0.22] ***	0.16	[0.07, 0.24] ***
Student status	0.04	[-0.06, 0.13]	0.07	[-0.03, 0.17]	-0.04	[-0.13, 0.05]	-0.07	[-0.16, 0.03]
Social status childhood	-0.06	[-0.15, 0.02]	-0.07	[-0.16, 0.02]	0.06	[-0.02, 0.14]	0.07	[-0.02, 0.15]
Social connectedness	-0.18	[-0.28, -0.08] ***	-0.25	[-0.35, -0.15] ***	0.33	[0.24, 0.42] ***	0.41	[0.31, 0.50] ***
**Criterion A**	-0.53	[-0.63, -0.43] ***	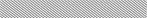		0.49	[0.39, 0.58] ***	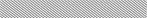	
**Mentalization**	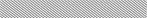		-0.41	[-0.51, -0.31] ***	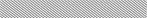		0.35	[0.26, 0.45] ***
*R*^2^ adj.	0.45***		0.38***		0.51***		0.44***	

DS, depressive symptoms; LS, life satisfaction; *p < 0.05, ***p < 0.001, n = 316; gender: 1 = male, 2 = female; and student status: 1 = student, 2 = no student.

Statistical analysis with Criterion A as a predictor showed a highly significant association with both outcome variables (depressive symptoms, Model A1 and life satisfaction Model, B1) in the expected direction. In addition, we also found a highly significant association between mentalization as a predictor and depressive symptoms (Model A2) and life satisfaction (Model B2) as outcome. Thus, participants with lower capacities in Criterion A and mentalization reported higher depressive symptoms and, conversely, participants who scored high in Criterion A and MZQ showed higher life satisfaction. In line with the current literature, we found a significant contribution of social connectedness predicting participant’s depressive symptoms and life-satisfaction. An increase in social connectedness was associated with lower depressive symptoms and a higher life satisfaction.

The results of the overall models of all regression analyses (Model A1 and A2 and Model B1 and B2) were statistically significant. Using Criterion A as main predictor participants′ depressive symptoms explained 45% of the variance [*F*(6, 309) = 44.44, *p* < 0.001, Model A1], and in addition, 51% of the variance in life-satisfaction [*F*(6, 309) = 56.42, *p* < 0.001, Model B1]. Moreover, overall models using mentalization as the main predictor variable reached again significance and explained 38% of the variance in depressive symptoms among the participants [*F*(6, 309) = 33.01, *p* < 0.001, Model B1], and 44% of the variance in participants’ life-satisfaction [*F*(6, 309) = 42.14, *p* < 0.001, Model B2].

Finally, we estimated a multivariate linear regression using Criterion A and mentalization as separate predictors and resilience as an outcome variable to test the eligibility of resilience as a mediator (Table 3). Again, all five covariates (age, gender, social status in childhood, student status, and social connectedness) were included. In the regression model with personality as a main predictor, resilience was positively associated with Criterion A (ß = 0.43, *p* < 0.001) and social connectedness (ß = 0.30, *p* < 0.001). Further analysis with mentalization as the main predictor showed mentalization (ß = 0.30, *p* < 0.001) and social connectedness (ß = 0.37, *p* < 0.001) as positive predictors. No other predictors reached significance. The overall model with Criterion A as the main regressor explained 40% of the variance in resilience among the participants [*F*(6, 309) = 35.71, *p* < 0.001]. Similarly, the overall model with mentalization explained 34% of the variance in participants’ resilience [*F*(6, 309) = 27.67, *p* < 0.001]. The results indicated that Criterion A and mentalization are relevant and almost comparable predictors in terms of the sign and value of the regression coefficients on resilience. Furthermore, it seems justified to include social connectedness as a control variable because of its strong association with resilience.

**TABLE 3:** Associations with resilience.

	Model A1 resilience			Model A2 resilience		
	B	SE (B)	β	B	SE (B)	β
Age	0.00	0.01	-0.04	0.00	0.00	0.03
Gender	0.02	0.09	0.01	0.04	0.09	0.02
Student status	0.10	0.10	0.05	0.06	0.10	0.03
Social status childhood	0.03	0.03	0.05	0.03	0.03	0.06
Social connectedness	0.59	0.10	0.30***	0.73	0.10	0.37***
**Criterion A**	0.78	0.09	0.43***	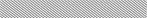		
**Mentalization**	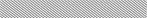			0.41	0.07	0.30***
*R*^2^ adj.	0.40***			0.34***		

***p < 0.001 and n = 316.

### Structural equation modeling

Structural equation modeling was applied to test the mediating role of resilience in the impact of mentalization and Criterion A on depressive symptoms and life satisfaction as indices of mental health. Similar to the linear regression analyses, we calculated two different models differing only in the predictor variable (SEM1 = Criterion A, SEM2 = mentalization). Since student status and social status in childhood were not significantly associated with the outcomes in the regression analysis, they were omitted in these models.

#### Measurement model

In both measurement models we included four latent variables (resilience, depressive symptoms, life satisfaction and Criterion A for SEM1 or mentalization for SEM2) and 12 observed variables. The models showed acceptable fit to the data (SEM1: *X*^2^ = 123.2, *df* = 48, *p* < 0.001; RMSEA = 0.071; SMR = 0.037; and CFI = 0.972 and SEM2: *X*^2^ = 115.8, *df* = 48, *p* < 0.001; RMSEA = 0.067; SMR = 0.039; and CFI = 0.974). All factor loadings on the latent variables reached significance (*p* < 0.001), indicating that all latent variables were well represented by their respective observed variables.

#### Structural model

**FIGURE 2:**
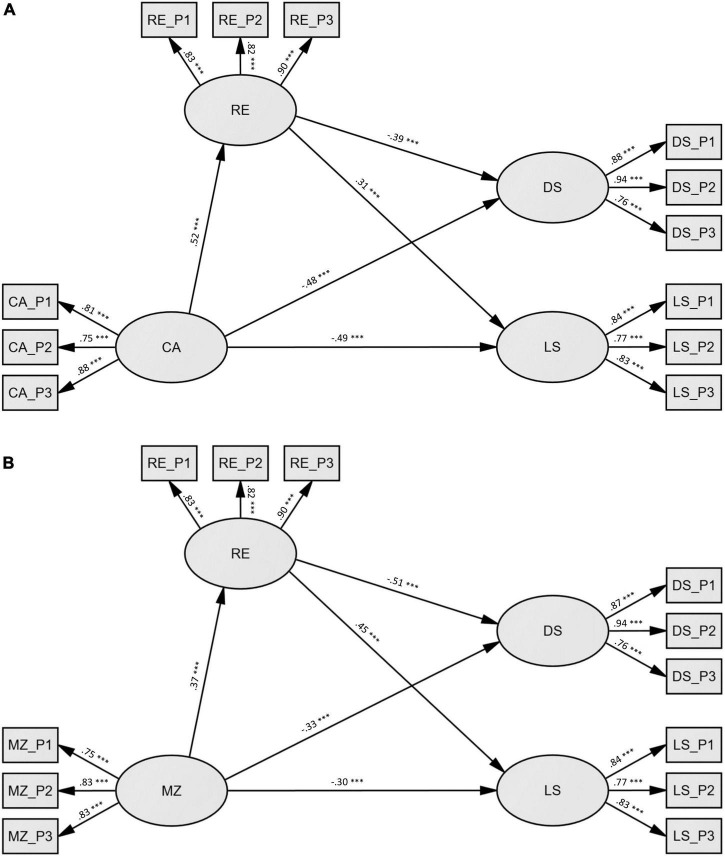
Illustration of structural models SEM1 and SEM2 (*n* = 316). **(A)** SEM1: independent variable = criterion A (CA), dependent variables = depressive symptoms (DS), and life-satisfaction (LS), mediator = resilience (RE). **(B)** SEM2: independent variable = mentalization (MZ), dependent variables = depressive symptoms (DS), and life-satisfaction (LS), mediator = resilience (RE). Both models showing standardized regression coefficients β and are controlled for age, gender, and social connectedness. All effects were significant at ***p < 0.001. CA_P1 – CA_P2 = three parcels of criterion A, MZ_P1 – MZ_P2 = three parcels of mentalization, RE_P1 – RE_P2 = three parcels of resilience, DS_P1 – DS_P2 = three parcels of depressive symptoms, LS_P1 – LS_P2 = three parcels of life satisfaction.

Both models were composed of 2 direct paths (predictor → depressive symptoms; predictor → life satisfaction) and 2 indirect paths (predictor → resilience → depressive symptoms; predictor → resilience → life satisfaction). Since we found strong associations between social connectedness and the other variables in the models, we added social connectedness, beside age and gender as covariates in the structural models to control for possible influences. The results of the structural modeling indicated an acceptable fit to the data for both models (SEM1: *X*^2^ = 186.3, *df* = 76, *p* < 0.001; RMSEA = 0.068; SMR = 0.043; and CFI = 0.962/SEM2: *X*^2^ = 192.8, *df* = 76, *p* < 0.001; RMSEA = 0.070; SMR = 0.048; and CFI = 0.958). Standardized path coefficients can be seen in Figure 2. Bootstrapping was used to test the significance of total, direct and indirect effects which are summarized in Table 4. Confidence intervals not containing zero indicated significant (*p* < 0.05) effects. Thus, as can be seen in Table 4, all direct, indirect, and total effects reached significance. A higher score on Criterion A/mentalization indicates higher capacities.

**TABLE 4:** Total, direct and indirect effects of SEM1 (Criterion A) and SEM2 (mentalization) on depressive symptoms and life satisfaction with resilience as mediator.

	Std. total effect		Std. direct effect		Std. indirect effect		Result
	Point est.	95% CI	Point est.	95% CI	Point est.	95% CI	
**SEM 1/Criterion A**							
Depressive symptoms	-0.68	[-0.80, -0.58]	-0.48	[-0.62, -0.33]	-0.20	[-0.30, -0.13]	Partial mediation
Life satisfaction	0.65	[0.53, 0.77]	0.49	[0.34, 0.63]	0.16	[0.10, 0.26]	Partial mediation
**SEM 2/Mentalization**							
Depressive symptoms	-0.52	[-0.64, -0.40]	-0.33	[-0.46, -0.21]	-0.19	[-0.28, -0.12]	Partial mediation
Life satisfaction	0.46	[0.32, 0.60]	0.30	[0.16, 0.44]	0.16	[0.10, 0.25]	Partial mediation

CI, confidence interval; Std., Standardized; est., estimate; *n* = 316; bootstrapping sample size = 2,000; controlled for age, gender, and social connectedness.

Structural equation modeling 1 – Path Model Criterion A (Figure 2A): First, we looked at the total effects. Results showed a large negative total effect on depressive symptoms (β = −0.68, 95% – CI [−0.80, −0.58]) and large negative total effect on life satisfaction (β = 0.65, 95% – CI [0.53, 0.77]). The mediation model for depression was divided into a moderate negative direct effect (β = −0.48, 95% – CI [−0.62, −0.33]) as well as a small negative indirect effect (β = −0.20, 95% – CI [−0.30, −0.13]) mediated *via* resilience. The mediation model for life satisfaction was divided into a moderate positive direct effect (β = 0.49, 95% – CI [0.34, 0.63]) as well as a low positive indirect effect (β = 0.16, 95% – CI [0.10, 0.26]) mediated *via* resilience. The direct effect showed on the path between Criterion A and the mediator resilience (β = 0.52, 95% – CI [0.40, 0.64]) a significant large effect and on the path between the mediator to depressive symptoms (β = −0.39, 95% – CI [−0.53, −0.24]) a negative moderate effect and to life satisfaction a positive moderate effect (β = 0.31, 95% – CI [0.17, 0.44]). To sum up, as expected, individuals with higher Criterion A scores are generally more resilient and satisfied in life and are less depressed. Higher levels of life satisfaction and lower depressive symptoms predicted by Criterion A were partly mediated through resilience.

Structural equation modeling 2 – Path Model Mentalization (Figure 2B): First, we again looked at the total effects, which showed a large negative effect on depressive symptoms (β = −0.52, 95% – CI [−0.64, −0.40]) and in contrast to Criterion A which revealed a large positive effect, only a moderate positive effect on life satisfaction (β = 0.46, 95% – CI [0.32, 0.60]). The mediation model for depression was divided into a moderate negative direct effect (β = −0.33, 95% – CI [−0.46, −0.21]) as well as a small negative indirect effect (β = −0.19, 95% – CI [−0.28, −0.12]) mediated *via* resilience. The mediation model for life satisfaction was divided into a small positive direct effect (β = 0.30, 95% – CI [0.16, 0.44]) as well as a small positive indirect effect (β = 0.16, 95% – CI [0.10, 0.25]) mediated *via* resilience. Regarding the direct effects, we found a significant moderate effect between the path mentalization and the mediator resilience (β = 0.37, 95% – CI [0.23, 0.50]), a large negative effect on the path between mediator and depressive symptoms (β = -0.51, 95% – CI [-0.63, -0.37]), and a moderate positive effect between mediator and life satisfaction (β = 0.45, 95% – CI [0.32, 0.58]).

To summarize, the results of the mentalization structural equation model (SEM2) showed comparable results to the model with Criterion A (SEM1) in terms of path coefficient and total, direct, and indirect effects. As predicted, higher personality functioning (Criterion A and mentalization) was associated with higher resilience and life satisfaction scores and lower depressive symptoms. However, the effect of Criterion A and mentalization on depressive symptoms and life satisfaction decreased but remained significant when the effect of the mediator (resilience) was considered in the analysis. This suggests that resilience may be only one of the factors involved in mediating the effect of personality on the outcomes. Furthermore, the results are in line with our results revealed by regression analyses.

## Discussion

Several studies have provided evidence on the impact of personality functioning (measured as Criterion A or mentalizing capacity) on pandemic-related effects on mental health during the COVID-19 pandemic (Lassri and Desatnik, 2020; Poulios et al., 2021; Ventura Wurman et al., 2021; Kvarstein et al., 2022; Yatziv et al., 2022). In our sample, higher depressive symptoms rates were measured when compared to normative values before the pandemic (Huang and Zhao, 2020). This suggests that the pandemic was a significant stressor in our sample. In contrast, personality functioning rates were comparable within published population norms (Spitzer et al., 2021).

In the current study, we focused on two research questions. First, we wanted to investigate the predictive capacity of either Criterion A and mentalization on mental health outcomes. Our modeling strategy was to use differing but related assessments of personality functioning (Criterion A in the AMPD, Spitzer et al., 2021, and mentalization Hausberg et al., 2012) as predictors of mental health outcomes to verify the robustness of conclusions to variations in the personality measures. Likewise, we considered two mental health outcomes (depressiveness, life satisfaction) in individuals from the general population to verify the extent to which the predictive capacity of personality functioning could be generalized. We found that the effects of personality functioning on mental health outcomes were similar irrespective of how functioning (Criterion A or mentalization capacity) or mental health outcomes were assessed. These findings are consistent with the results of previous studies on the mental health outcomes associated with personality functioning conducted prior to the pandemic (e.g., Taubner et al., 2011; Fischer-Kern and Tmej, 2019; Dagnino et al., 2020; Schwarzer et al., 2021a).

In our models, these two predictors were correlated among participants, were fitted by comparable coefficients, and explained a similar portion of the variance. Our results are therefore in line with the evidence reported by Zettl et al. (2020) on the operational overlap of these two constructs, which led these authors to conclude that they provide a largely equivalent assessment of personality functioning. Prior to that study, several researchers had noted that both constructs capture important phenomena relevant to clinical and general mental health issues (Bender et al., 2011; Waugh, 2019; Buer Christensen et al., 2020; Fonagy and Campbell, 2021). Nevertheless, they also appear to differ in that Criterion A aims to capture a broader set of personality self-other functions. Mentalization specifically refers to an individual’s ability to become aware of one own′s intentions, desires, thoughts, and feelings and to perceive others as beings with these mental states. In contrast, the self-other domains of Criterion A encompass four human core capacities: identity, self-direction, empathy, and intimacy. Among these, identity and empathy were thought to be associated with mentalizing (Bender et al., 2011). However, Zettl et al. (2020) found that all subscales of the LPFS-BF (which assesses Criterion A) were associated with mentalization. Our findings suggest that further research is needed to clarify the relationship between these two constructs and highlight the importance of refining our assessment instruments.

A second aim of the present study was to test the possible role of resilience as a mediator on the effects of Criterion A and mentalizing capacity as predictors of mental health outcomes (depressive symptoms and life satisfaction) during the COVID-19 pandemic in a population sample. The hypothesis of resilience as mediator of the positive effects of personality functioning on mental health outcomes has been formulated by Fonagy et al. (2017a) but has so far been scarcely explored empirically. Using structural equation modeling, we found that resilience was a significant mediator of the effects of Criterion A/mentalizing on both outcomes, confirming the hypothesis that higher resilience may account at least in part for the effects of higher personality functioning on mental health outcomes. This finding is consistent with the theoretical formulation about the roots of resilience in aspects of psychic functioning related to mentalization, such as “epistemic trust” (Fonagy et al., 2017a,b).

Against the theoretical background of these two instruments, the results concerning personality functioning would therefore imply that individuals with stable self- and interpersonal functioning or self-other representations are able to flexibly integrate the impacts of the external world and internal demands into their own in order to adapt to current circumstances. In individuals with low functioning, by contrast, distressing events may be likely to be perceived as disorganizing due to an inaccurate or non-mentalized representation of the situation and possible actions that could be taken (Gergely et al., 2002; Fonagy et al., 2017a,b; Fonagy and Luyten, 2018). These authors proposed that relatively automatic, reflexive processes are associated with less sophisticated, impulsive actions and rigid interpersonal behavior, whereas regulated, cooperative, and goal-directed behaviors are associated with a reflective mode (Viviani et al., 2011; Fonagy et al., 2017b; Labek et al., 2019).

Focusing on personality functioning abilities is of particular interest because it may be improved by interventions that focus on specific self-other domains (Young et al., 2003; Bateman and Fonagy, 2004; Kernberg et al., 2008; Hopwood, 2018; Fonagy et al., 2019), which are an important component of all psychotherapies of severe PD (Bender et al., 2011; Viviani et al., 2011). Several treatments have already demonstrated their therapeutic efficacy in PD, such as Mentalization-Based Treatment (Bateman and Fonagy, 2004), Transference-Focused Psychotherapy (Kernberg et al., 2008), Schema-Focused Psychotherapy (Young et al., 2003), and Dialectical Behavior Therapy (Linehan, 2020). An open question is whether such interventions may also be of preventive value in subclinical populations in time of stress like the COVID-19 pandemic.

## Summary and conclusions

First, our results support Zettl et al.’s (2020) findings that Criterion A and mentalizing share substantial overlaps conceptually and empirically. Second, in addition, our results foster Fonagy’s assumptions that mentalizing serves as a protective function by promoting resilience to the impact of stress and threats. Third, our results strengthen the contention of other authors that personality functioning may be a key transdiagnostic factor (Krueger and Eaton, 2015; Waugh, 2019; Buer Christensen et al., 2020) relevant to both psychopathology and positive health outcomes.

In conclusion, we suggest that it may be fruitful for further research to explore personality functioning as resilience-promoting factor or mechanism and their possible influence not only on life satisfaction and depression, but also on psychological functioning in general. Whether personality functioning can be considered as a general factor in terms of severity indicating a core self-other vulnerability of human health still needs future research. However, there is compelling evidence that a dimensional approach has the potential to stimulate and improve research on mental health issues as well as attempts to assess, prevent, and treat mental illness more adequately.

## Scope and limitations

The present study is affected by several limitations. First, its scope is limited since a large proportion of participants were young students in their 20s and women were generally overrepresented. Personality functioning was comparable to population values, but this group of participants may have reacted to the restrictions during the pandemic with increased distress. Second the study was conducted with a cross-sectional design. Only a longitudinal design would have allowed comparing the effects of the pandemic with pre-pandemic data; here, we had to rely on comparison with population normative data to assess the effect of the pandemic on mental health. Third, the use of self-report measures for assessing Criterion A and mentalizing capacities may have led to over-emphasizing consciously available information at the expense of more implicit aspects of these constructs. Fourth, to avoid bias, the mediator variable resilience must be controlled. Hence, we included social connectedness as a confounding covariate in the models, as social connectedness is a known predictor of resilience. However, we cannot exclude the existence of other possible confounders for resilience, which may have led to overestimating the extent of the mediation. Finally, associations between rating scales should be considered with caution, as they may ensue from semantic similarities in the questions asked to participants.

## Data availability statement

The raw data supporting the conclusions of this article will be made available by the authors, without undue reservation.

## Ethics statement

The study is in accordance with the Declaration of Helsinki and was reviewed and approved by the Review Board for ethical questions in scientific research of the Institute of Psychology of the University of Innsbruck (N° 34/2020) before data were collected. Written informed consent to participate in this study was provided by the participants.

## Author contributions

KL and JM conceptualized the study, organized and conducted the study setup and data collection. JM, KL, and LR conducted the raw data analysis. JM, KL, and RV conducted the statistical data modeling. AB and RV provided the important intellectual contributions in commenting and revising the manuscript. KL, JM, AB, RV, LR, and DR wrote the manuscript and edited its final version. All authors contributed to the article and approved the submitted version.

## Conflict of interest

The authors declare that the research was conducted in the absence of any commercial or financial relationships that could be construed as a potential conflict of interest.

## Publisher’s note

All claims expressed in this article are solely those of the authors and do not necessarily represent those of their affiliated organizations, or those of the publisher, the editors and the reviewers. Any product that may be evaluated in this article, or claim that may be made by its manufacturer, is not guaranteed or endorsed by the publisher.
